# Atrial Electrogram Fractionation Distribution before and after Pulmonary Vein Isolation in Human Persistent Atrial Fibrillation—A Retrospective Multivariate Statistical Analysis

**DOI:** 10.3389/fphys.2017.00589

**Published:** 2017-08-24

**Authors:** Tiago P. Almeida, Gavin S. Chu, Xin Li, Nawshin Dastagir, Jiun H. Tuan, Peter J. Stafford, Fernando S. Schlindwein, G. André Ng

**Affiliations:** ^1^Department of Engineering, University of Leicester Leicester, United Kingdom; ^2^Biomedical Engineering, Center for Engineering, Modelling and Applied Social Sciences, Federal University of ABC São Bernardo do Campo, Brazil; ^3^Department of Cardiovascular Sciences, University of Leicester Leicester, United Kingdom; ^4^University Hospitals of Leicester NHS Trust Leicester, United Kingdom; ^5^National Institute for Health Research Leicester Cardiovascular Biomedical Research Centre, Glenfield Hospital Leicester, United Kingdom

**Keywords:** catheter ablation, atrial fibrillation, pulmonary veins, atrial electrograms, multivariate analysis, fractionation

## Abstract

**Purpose:** Complex fractionated atrial electrograms (CFAE)-guided ablation after pulmonary vein isolation (PVI) has been used for persistent atrial fibrillation (persAF) therapy. This strategy has shown suboptimal outcomes due to, among other factors, undetected changes in the atrial tissue following PVI. In the present work, we investigate CFAE distribution before and after PVI in patients with persAF using a multivariate statistical model.

**Methods:** 207 pairs of atrial electrograms (AEGs) were collected before and after PVI respectively, from corresponding LA regions in 18 persAF patients. Twelve attributes were measured from the AEGs, before and after PVI. Statistical models based on multivariate analysis of variance (MANOVA) and linear discriminant analysis (LDA) have been used to characterize the atrial regions and AEGs.

**Results:** PVI significantly reduced CFAEs in the LA (70 vs. 40%; *P* < 0.0001). Four types of LA regions were identified, based on the AEGs characteristics: (i) fractionated before PVI that remained fractionated after PVI (31% of the collected points); (ii) fractionated that converted to normal (39%); (iii) normal prior to PVI that became fractionated (9%) and; (iv) normal that remained normal (21%). Individually, the attributes failed to distinguish these LA regions, but multivariate statistical models were effective in their discrimination (*P* < 0.0001).

**Conclusion:** Our results have unveiled that there are LA regions resistant to PVI, while others are affected by it. Although, traditional methods were unable to identify these different regions, the proposed multivariate statistical model discriminated LA regions resistant to PVI from those affected by it without prior ablation information.

## Introduction

Pulmonary vein isolation (PVI) has been proven effective in the treatment of paroxysmal atrial fibrillation (Haissaguerre et al., 2000), but insufficient for persistent atrial fibrillation (persAF) due to patient specific structural and electrical changes in the left atrium (LA; Brooks et al., 2010). Atrial electrograms (AEGs) with low amplitude and multiple deflections/activations were thought to represent cardiac tissue with structural and electric remodeling induced by sustained atrial fibrillation (AF). Complex fractionated atrial electrograms (CFAEs) have been introduced as markers of such atrial sites and as potential targets for ablation (Nademanee et al., 2004). The high curative success rates reported in early data helped to consolidate CFAE-guided ablation as an adjunctive therapy to PVI for persAF (Calkins et al., 2012). The results reported in more recent investigations, however, have cast doubt on the efficacy of this approach. While some studies confirmed benefits of CFAE-guided ablation (Deisenhofer et al., 2009; Verma et al., 2010; Seitz et al., 2016), others concluded it is ineffective (Oral et al., 2009; Verma et al., 2015). Similarly, recently introduced Focal Impulse and Rotor Modulation (FIRM)-guided ablation is also being questioned. Early data have suggested high success rates in persAF treatment (Narayan et al., 2012), but recent studies have failed to confirm those results (Mohanty et al., 2016). Additionally, recent works have shown that endo-epicardial asynchrony may play a major role in the pathophysiology of AF and may offer an explanation why therapy fails in some patients (de Groot et al., 2010, 2016; Eckstein et al., 2011; Hansen et al., 2015). In such cases, the presence of focal fibrillation waves during AF can, besides ectopic activity, be explained by asynchronous activation of the atrial endo- and epicardial layer and transmurally propagating fibrillation waves (de Groot et al., 2016). The existence of different theories to explain AF mechanisms has led to intense debate regarding the strategy for persAF ablation, and a more thorough characterization of the underlying LA tissue might help in the identification of targets for persAF ablation.

Previous works have reported that CFAE distribution was affected by PVI, which motivated AEG-guided ablation after PVI (Roux et al., 2009; Tuan et al., 2011; Dixit et al., 2012). Ablation target identification relies on few—if not only one—descriptors measured from the AEGs for atrial tissue characterization (Deisenhofer et al., 2009; Verma et al., 2010, 2015; Dixit et al., 2012). Additionally, current guidelines for CFAE mapping performed after PVI do not consider the characteristics of the underlying LA tissue prior to PVI. These might represent limiting factors for a thorough understanding of the underlying atrial substrate.

In the present study, we sought to investigate atrial regions resistant to PVI and roof line ablation (PVI+RL) and atrial regions that are affected by it. These regions (resistant or affected by PVI−RL) could represent different AF drivers, and their discrimination might improve ablation target identification in persAF. Since single attributes calculated by the commercial systems might not be sufficient to discriminate those regions, multivariate statistical models based on multiple attributes measured from the AEGs were created to better characterize those regions.

## Materials and methods

### Electrophysiological study

Eighteen persAF patients (16 male; mean age 56.1 ± 9.3 years; history of AF 67.2 ± 45.6 months) referred to our institution for first time catheter ablation were included in this study (Tuan et al., 2011). Anti-arrhythmic and rate-controlling drugs (other than amiodarone) were stopped at least 5 half-lives before the procedure. Details of the clinical characteristics of the study subjects are provided in Table 1. All procedures were performed with full informed consent.

**Table 1:** Clinical characteristics of study population (*N* = 18).

Age, yrs.	56.1 ± 9.3
Male/Female	16/2
History of AF, months	67.2 ± 45.6
Ejection fraction, %	48 ± 1
Left atrial diameter, mm	47 ± 1
History of coronary artery disease	4
Medication^*^ (number of patients on)	
ACE inhibitor/ARB	11
Amiodarone	10
Beta-blockers	8
Calcium channel blockers	2
Digoxin	1
Sotalol	5

*Values are mean ± SD or n*.

* *ACE, angiotensin-converting-enzyme; AF, atrial fibrillation; ARB, angiotensin receptor blockers*.

The 3D LA anatomy was created within Ensite NavX^TM^ (St. Jude Medical, St. Paul, MN, USA) using a deflectable, variable loop circular mapping catheter (Inquiry Optima, St. Jude Medical). PVI was performed with a point-by-point wide area circumferential ablation approach, followed by the creation of a single roof line (Cool Path Duo irrigated RF catheter, St. Jude Medical).

No additional ablation targeting CFAE was performed in this study. Sequential point-by-point bipolar AEGs were collected from pre-determined atrial regions before and after PVI also using the variable loop circular mapping catheter Inquiry Optima (4.5 mm inter-electrode distance, 2 mm tip electrode for distal position, 10 poles) (Tuan et al., 2011). All patients were in AF before and after PVI+RL during signal collection.

### Signal analysis

A total of 797 AEGs were recorded from the LA, 455 before and 342 after PVI+RL, with a sampling frequency of 1,200 Hz, and band-pass filtered within 30–300 Hz. The AEGs, their corresponding CFE-Mean, CFE-StdDev and peak-to-peak (PP) values were exported from NavX with a fixed time window length of 2.5 s. A validated offline MATLAB algorithm was used to obtain the interval confidence level (ICL), the average complex interval (ACI) and the shortest complex interval (SCI), as defined by CARTO (Biosense Webster, Diamond Bar, CA, USA) (Almeida et al., 2016).

**Figure 1:**
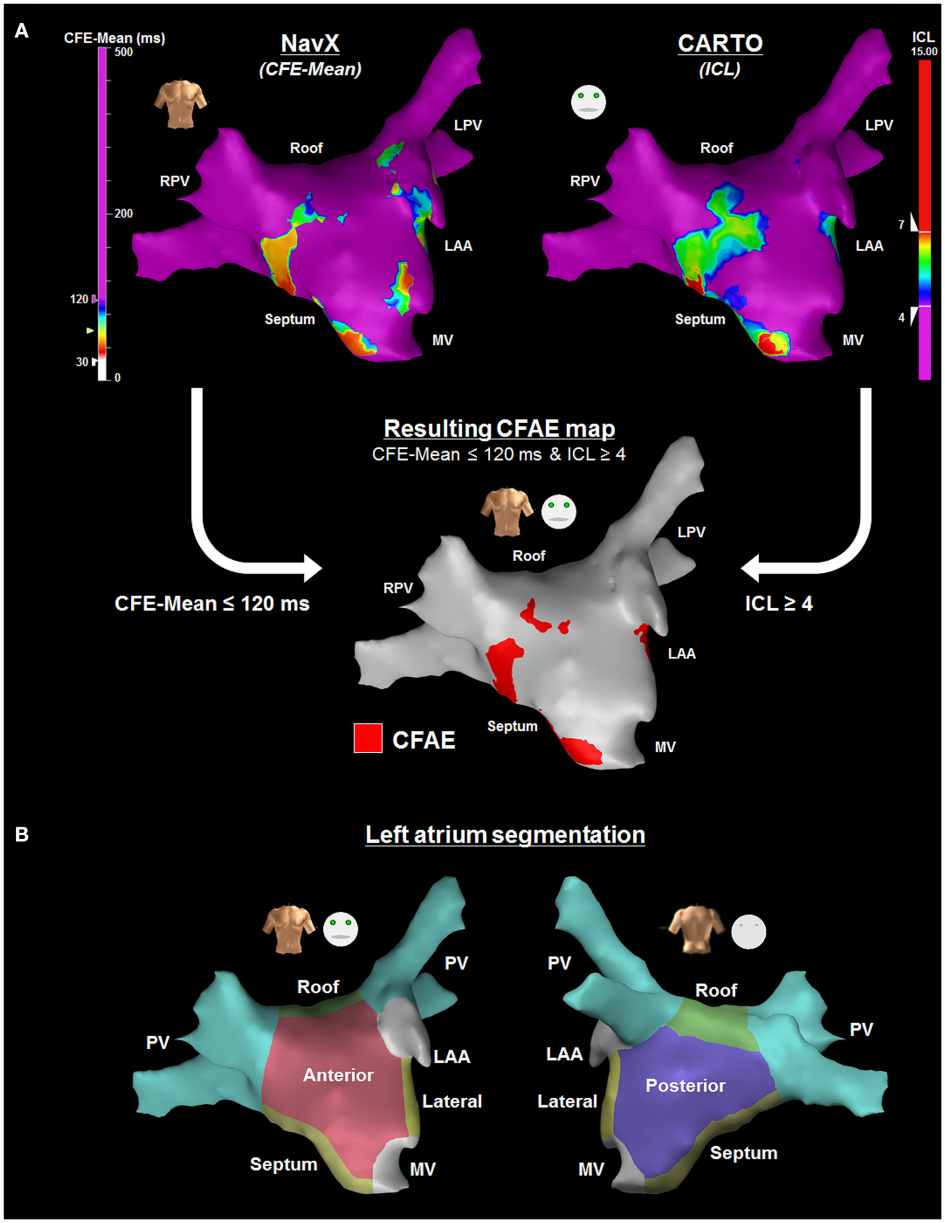
**(A)** CFAE definition as defined by both NavX and CARTO systems were considered in the current study—NavX (upper left) and CARTO (upper right) 3D LA anatomy representation for the same patient, with their respective automated CFAE detection algorithms (Almeida et al., 2016). LA regions hosting AEGs with both CFE-Mean ≤ 120 ms and ICL ≥ 4 were considered CFAEs (bottom map). **(B)** Illustration of the left atrium segmentation performed visually by an experienced clinician following a six-segment model encompassing the PVs; Roof; Posterior; Anterior; Septum and Lateral regions—anterior view (left) and posterior view (right).

CFAE criteria as defined by both systems were considered in the current study, in an attempt to minimize the inconsistencies between the two methods. CFAEs were defined as AEGs with both CFE-Mean ≤ 120 ms and ICL ≥ 4 (Figure 1A). The anatomical location of each AEG was visually classified by an experienced clinician using current clinical guidelines (Ho et al., 2012), following a six-segment LA model (as illustrated by Figure 1B), encompassing the PVs; Roof; Posterior; Anterior; Septum and Lateral regions.

### Multiple AEG attributes

Many methods have been developed to address AF drivers (Everett et al., 2001; Sanders et al., 2005; Oakes et al., 2009; Narayan et al., 2012; Ganesan et al., 2013; Almeida et al., 2016). In the present study, we have considered 12 relevant attributes measured from the AEGs used in previous studies as markers for AF drivers, accordingly:

*Attributes measured by commercial mapping systems*: CFE-Mean and CFE-StdDev, as defined by NavX. AEGs with CFE-Mean within 30–120 ms are considered fractionated and CFE-Mean > 120 ms represents low fractionation; ICL, ACI and SCI, as defined by CARTO; Typically, ICL < 4 represents low fractionation, 4 ≤ ICL < 7 refers to moderate fractionation and ICL ≥ 7 indicates high fractionation (Almeida et al., 2016).

CFE-Mean and CFE-StdDev measure the overall fractionation interval of AEGs based on negative deflections, while ICL, ACI, and SCI help to characterize complex intervals considering peaks and troughs. These attributes, measured by commercial mapping systems, have been extensively used to guide persAF ablation with varying outcomes (Deisenhofer et al., 2009; Oral et al., 2009; Verma et al., 2010, 2015; Dixit et al., 2012; Kim et al., 2017).

*Information theory attributes*: Shannon entropy (ShEn), sample entropy (SampEn), and Kullback-Leibler (K-L) divergence. Both ShEn and SampEn have high values for CFAEs, while K-L has small values for CFAEs (Shannon, 1948; Cover and Thomas, 1991; Lake et al., 2002). Information theory attributes provide a direct estimation of the amplitude distribution of a signal and, therefore, its complexity (Narayan et al., 2012; Ganesan et al., 2013). Entropy, for instance, has been reported to correlate with the core of rotors during FIRM mapping (Ganesan et al., 2013), which could contribute to electrophysiological studies targeting re-entries during ablation (Narayan et al., 2012).

*Amplitude based attributes*: PP and amplitude root mean square (RMS). Low voltage zones (LVZ) have been shown to correlate with atrial substrate (Oakes et al., 2009). PP and RMS might help to identify LVZs related with scar cardiac tissue, and ablating those has been shown to be a promising AF ablation strategy (Oakes et al., 2009; Schade et al., 2016).

*Frequency based attributes*: dominant frequency (DF) and organization index (OI). OI is bounded between 0 and 1, and smaller values indicate more fractionated AEGs (Everett et al., 2001; Sanders et al., 2005). Frequency analysis helps to identify atrial regions with high activation rate during AF. It has been suggested that ablation at these sites could be an effective way to organize AF (Sanders et al., 2005).

A brief review on these attributes is provided in the Supplementary Materials.

### Statistical analysis

All non-normally distributed variables are expressed as median ± interquartile range (IQR). Nonparametric paired multiple data were analyzed using the Friedman test with Dunn's correction. Spearman's correlation was computed to quantify the correlation between the attributes. Multivariate statistical models were created with robust multivariate analysis of variance (MANOVA) using Munzel and Brunner's method (Munzel and Brunner, 2000), and linear discriminant analysis (LDA) with the 12 attributes. The discriminant scores for the LDA were calculated with the entire database, while the model was validated with the leave-one-out cross-validation (LOOCV) method. Two statistical models were created for two sets of data, separately: before and after PVI+RL. *P* < 0.05 were considered statistically significant.

## Results

From the 455 AEGs collected before and 342 AEGs collected after PVI+RL, 207 pairs of AEGs (before and after PVI+RL) were found to be recorded at similar LA locations (i.e., 207 LA locations: 207 AEGs collected before and 207 AEGs collected after PVI+RL). Only these 207 locations (~12 locations per patient) were used for the remaining parts of the study to allow for the investigation of the local impact of PVI+RL in patients with persAF. Changes in atrial volume and shifts in the 3D LA anatomy reconstructed by commercial systems are common before and after ablation, which can distort the reconstructed LA map. Therefore, the points collected before and after PVI+RL were considered to belong to similar locations if the points were spaced by no more than 5 mm apart from each other to compensate for this limitation. Only points remote from ablation lesions were considered in the analyses.

Out of the 207 LA locations, 4 (2%) were collected at the PVs; 20 (10%) at the Roof; 59 (29%) at the Posterior; 84 (40%) at the Anterior; 25 (12%) at the Septum; and 15 (7%) at the Lateral.

### Effect of PVI+RL on the distribution of CFAEs

**Figure 2:**
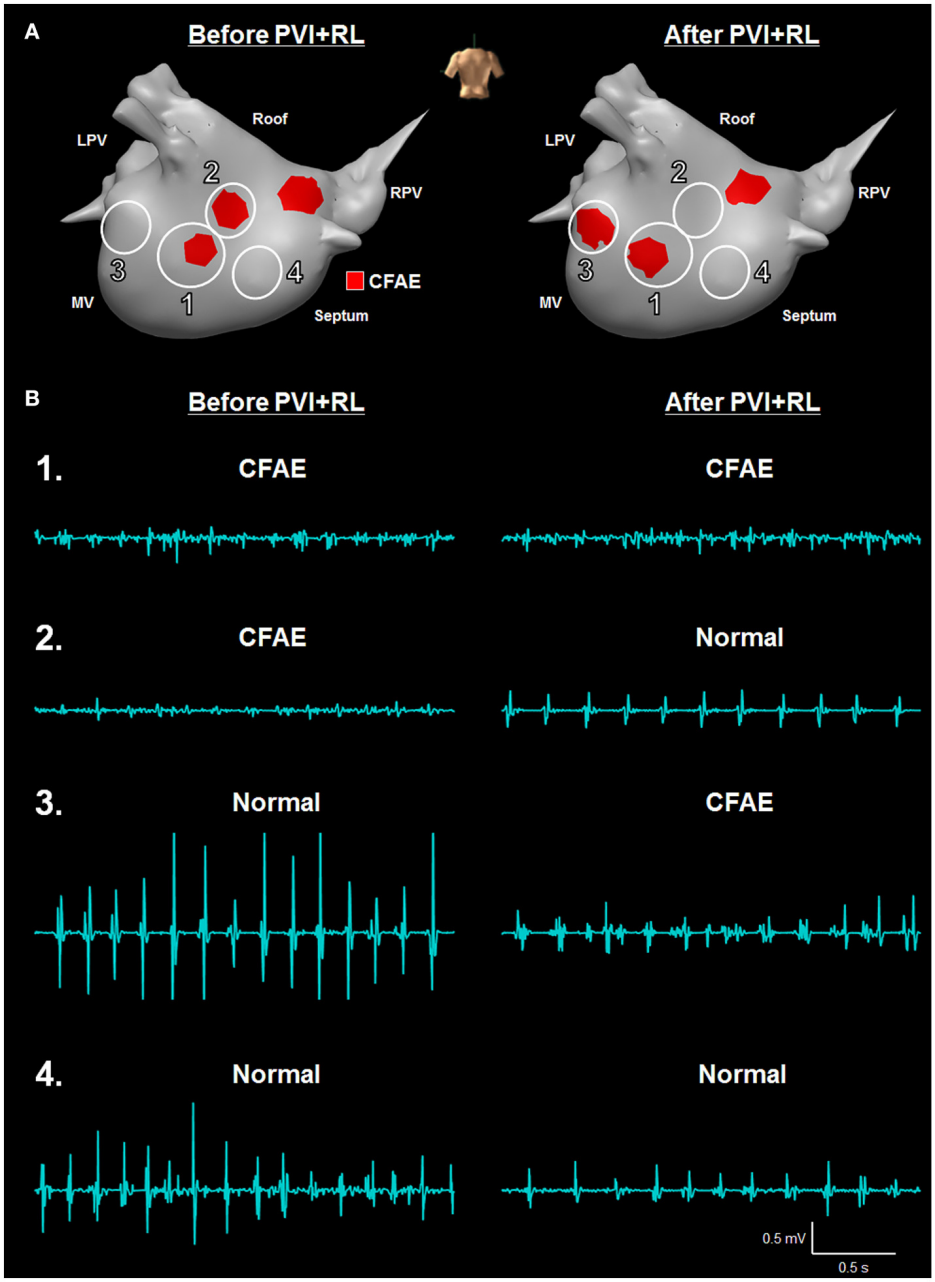
Illustration of the different types of LA regions found in this study—**(A)** Four groups of atrial regions were identified in terms of CFAE, before and after PVI+RL: CFAE before and after PVI+RL (group 1); CFAE before, Normal after PVI+RL (group 2); Normal before, CFAE after PVI+RL (group 3); and Normal before and after PVI+RL (group 4). **(B)** Illustration of AEGs found in each group. Fractionated AEGs were found in group 1 before and after PVI+RL; fractionated AEGs before PVI+RL with increased organization after PVI+RL in group 2; organized activation before PVI+RL with increased fractionation after PVI+RL in group 3; organized activation before and after PVI+RL in group 4.

The LA locations have been classified in four groups based on their AEG characteristics as before and after PVI+RL, as illustrated in Figure 2A: in group 1, the LA locations were classified as CFAEs at baseline and remained CFAEs after PVI+RL; in group 2, the LA locations were classified as CFAEs at baseline and converted to normal after PVI+RL; in group 3, the LA locations had normal AEGs at baseline and became CFAEs after PVI+RL; in group 4, the LA locations had normal AEGs at baseline and remained normal after PVI+RL. Figure 2B illustrates the AEGs found in each of the four groups.

**Figure 3:**
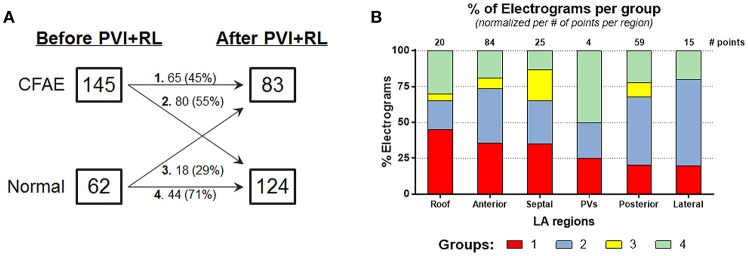
Effect of PVI on the AF behavior—**(A)** 45% of the AEGs classified as CFAEs before ablation remained fractionated after ablation, while the remainder became Normal. 71% of the AEGs classified as Normal before ablation remained not fractionated after ablation, while the remainder became CFAE. **(B)** Incidence of different types of AEGs (group 1, 2, 3, and 4) in the different LA regions, namely: roof, anterior wall, septum, PVs, posterior wall, and lateral. The number of paired points—before and after ablation—collected per regions is also provided.

Out of the 207 LA locations, 65 (31%) were classified as belonging in group 1; 80 (39%) in group 2; 18 (9%) in group 3 and; 44 (21%) in group 4. Figure 3A illustrates the occurrence of groups in the LA anatomical sites. Group 1 was observed in all sites, with the LA roof showing the highest incidence, followed by the anterior wall, septum, PVs, posterior wall and lateral LA. Group 2 was observed in all sites, with the lateral LA showing the highest incidence. The PVs and lateral LA were the only sites with no occurrence of group 3. LA regions with normal AEGs before and after PVI+RL (group 4) were also observed in all sites.

Additionally, our results show that PVI+RL reduced the overall LA fractionation. 70% of the LA locations were classified as CFAEs at baseline. After PVI+RL, the number of locations classified as CFAEs decreased to 40% (*P* < 0.0001). As illustrated in Figure 3B, 45% of the locations that were CFAEs before PVI+RL remained fractionated following PVI+RL (group 1, 31% of all 207 points), while 55% converted to normal (group 2, 39% of all 207 points); 29% of the normal AEGs prior PVI+RL became fractionated (group 3, 9% of all 207 points), while 71% remained normal (group 4, 21% of all 207 points).

### Correlation between AEG attributes

It has been previously suggested that poor correlation between attributes measured from AEGs during AF is a strong indicative that these attributes would represent a poor measure of the atrial substrate (Lau et al., 2015). Poor correlation between two (or more) attributes indicates that the attributes are measuring different aspects of an AEG. Therefore, poorly correlated attributes would provide further information about the AEG under analysis. Additionally, low correlation among attributes is usually desired for a proper multivariate classification.

**Figure 4:**
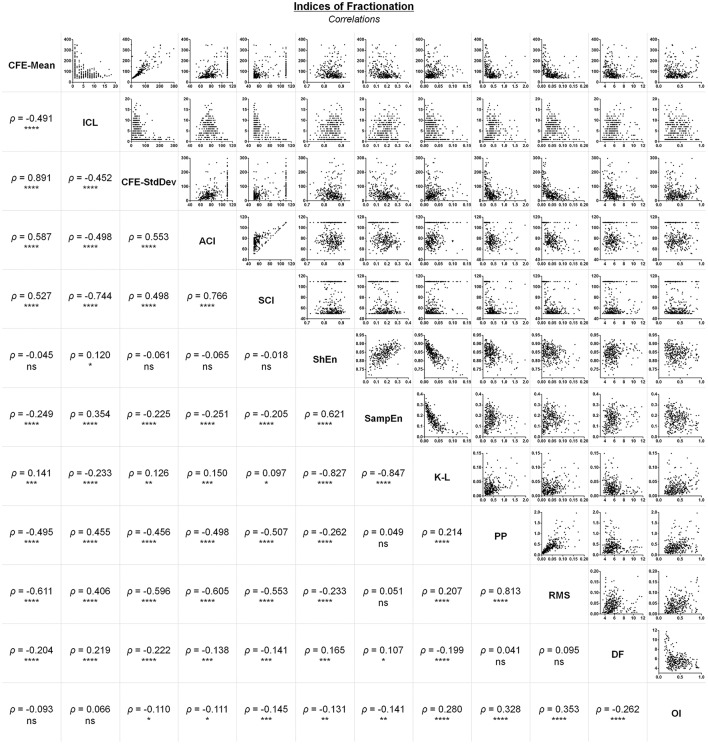
Correlations between different attributes measured from the same AEG database. ^****^P < 0.0001; ^***^P < 0.001; ^**^P < 0.01; ^*^P < 0.05; ns, not significant.

All attributes have been compared to each other as illustrated on Figure 4, with the Spearman's correlation coefficients between them. The attributes correlated poorly with each other, except attributes with biasing effects, which had shown high correlation between them, such as: CFE-Mean vs. CFE-StdDev (ρ = 0.891, *P* < 0.0001); ICL vs. SCI (ρ = −0.744, *P* < 0.0001); ACI vs. SCI (ρ = 0.766, *P* < 0.0001); ShEn vs. SampEn (ρ = 0.621, *P* < 0.0001); ShEn vs. K-L (ρ = −0.827, *P* < 0.0001); SampEn vs. K-L (ρ = −0.847, *P* < 0.0001); PP vs. RMS (ρ = 0.813, *P* < 0.0001).

The remaining attributes had moderate or poor correlation with each other, suggesting they would be ideal for multivariate classification.

### Effect of PVI+RL on AEG attributes

**Figure 5:**
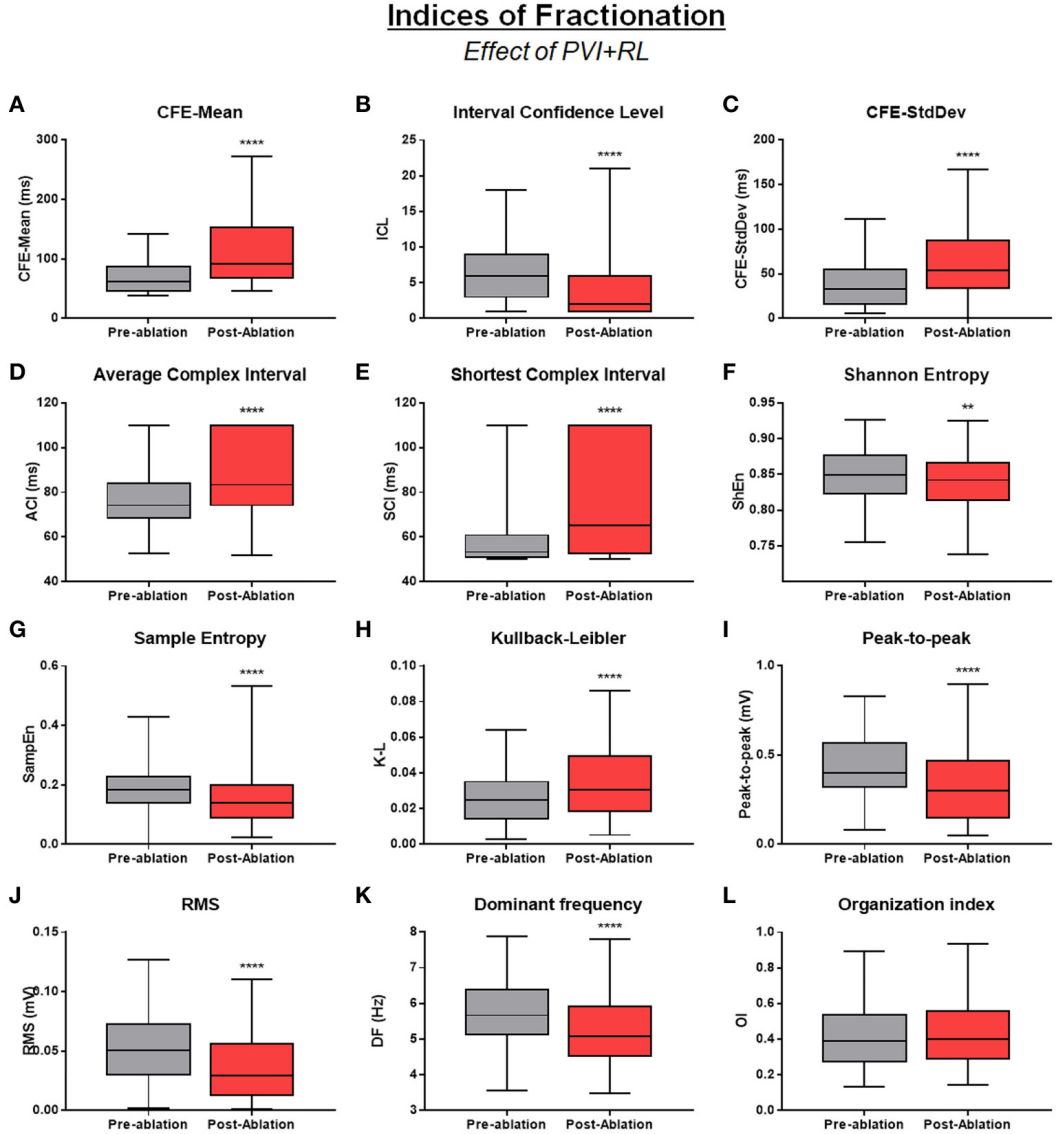
Effect of PVI+RL on the attributes—Effect of PVI and roof line creation on: **(A)** CFE-Mean; **(B)** ICL; **(C)** CFE-StdDev; **(D)** ACI; **(E)** SCI; **(F)** ShEn; **(G)** SampEn; **(H)** K-L; **(I)** PP; **(J)** RMS; **(K)** DF; **(L)** OI. All attributes, except OI, were significantly affected by ablation. All data are expressed as minimum, maximum and median ± IQR. ^****^P < 0.0001; ^**^P < 0.01.

Although, most AEG attributes correlated poorly with each other, ablation had a significant effect on all attributes, except for the OI (Figure 5).

All attributes measured by commercial mapping systems indicated that PVI+RL had reduced AEG fractionation. CFE-Mean, CFE-StdDev, ACI, SCI significantly increased after PVI+RL, while ICL significantly decreased after ablation (all *P* < 0.0001).

Information theory attributes, amplitude based attributes and frequency based attributes have been used in previous studies as markers for AF drivers. For instance, entropy has been reported to correlate with the core of rotors (Ganesan et al., 2013), which could contribute to investigations using the FIRM ablation strategy (Narayan et al., 2012); LA sites with low amplitude have been correlated with fibrotic tissue with low conduction velocity, participating in the perpetuation of the arrhythmia (Oakes et al., 2009); DF is related to the main activation wavefront in an AEG and might represent atrial regions hosting either rapid ectopic activities or small re-entry circuits that are driving the arrhythmia (Sanders et al., 2005). In the present work, information theory attributes and frequency based attributes suggested that PVI+RL have improved the activation organization as measured by the entropies (before vs. after PVI+RL; SampEn: 0.19 ± 0.06 vs. 0.15 ± 0.08, *P* < 0.0001; ShEn: 0.85 ± 0.05 vs. 0.84 ± 0.05, *P* = 0.005; K-L: 0.03 ± 0.01 vs. 0.04 ± 0.03, *P* < 0.0001) and reduced the atrial activation rate, as measured by the DF (6.06 ± 1.65 Hz vs. 5.45 ± 1.39 Hz, *P* < 0.0001). On the other hand, amplitude based attributes decreased after PVI+RL, suggesting the presence of extended fibrotic tissue (PP: 0.47 ± 0.28 mV vs. 0.36 ± 0.30 mV; RMS: 0.06 ± 0.04 mV vs. 0.04 ± 0.03 mV, both *P* < 0.0001).

### Multivariate statistical model for atrial substrate characterization before and after PVI+RL

As expected, CFE-Mean (*P* < 0.0001), CFE-StdDev (*P* < 0.0001), ACI (*P* < 0.0001), SCI (*P* < 0.0001), and K-L (*P* = 0.0004) were all lower for AEGs classified as CFAEs when compared to normal AEGs either before or after PVI+RL. Similarly, ICL (*P* < 0.0001), ShEn (*P* = 0.06), SampEn (*P* < 0.0001), PP (*P* < 0.0001), RMS (*P* < 0.0001), DF (*P* = 0.0044), and OI (*P* = 0.0086) demonstrated higher values for CFAEs. However, no single attribute on its own was able to discriminate the different LA region groups, whether measured before or after PVI+RL (further details in the Supplementary Materials).

**Figure 6:**
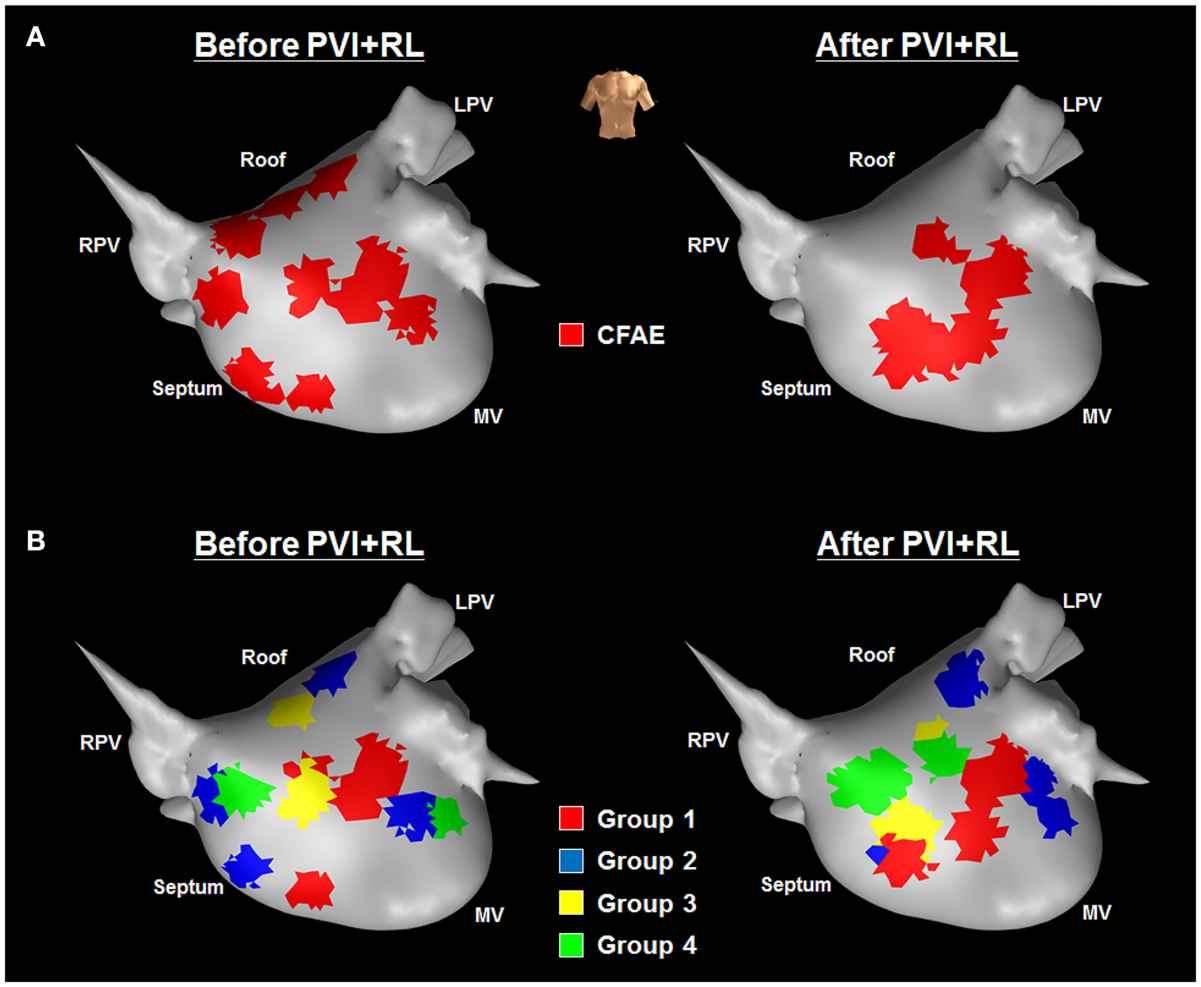
LA maps from one patient—before (left hand side) and after (right hand side) PVI+RL—illustrating the atrial regions marked according to **(A)** the commercial systems and **(B)** to the proposed method—**(A)** CFAE map generated by the commercial systems; **(B)** LA marked according to the multivariate model with the following regions: Group 1—CFAEs at baseline that remained CFAEs after PVI+RL; Group 2—CFAEs at baseline that converted to normal after PVI+RL; Group 3—normal AEGs at baseline that became CFAEs after PVI+RL; Group 4—normal AEGs at baseline that remained normal after PVI+RL.

MANOVA suggests a significant main effect of the LA region groups (1 to 4) on the attributes on both before (*F*-*ratio F* = 9.41, *P* < 0.0001) and after ablation (*F* = 14.74, *P* < 0.0001) datasets. MANOVA was followed up with LDA, which revealed three discriminant functions both before and after PVI+RL. The LOOCV revealed that the LDA successfully discriminated, prior to any ablation, 40% of the AEGs in group 1; 73% of group 2; 39% of group 3 and; 59% of group 4. After PVI+RL, LDA correctly identified 86% of the AEGs in group 1; 76% of group 2; 5.6% of group 3 and; 27% of group 4. The ranked results from MANOVA and the coefficients from the LDA are provided in the Supplementary Materials. Figure 6 illustrates the LA map from one patient with the atrial regions marked as CFAEs following the commercial systems criteria (Figure 6A), and the LA marked by the proposed model into the four groups (Figure 6B; group 1, 2, 3, and 4), before and after PVI+RL.

## Discussion

Our results, presented in this study, provide additional evidence that PVI changes the CFAE distribution in the LA of patients with persAF. However, we provide new evidence that some LA regions are resistant to PVI, while other regions are affected by it. Some CFAEs were resolved after PVI and became organized AEGs. On the other hand, some CFAEs persisted after PVI. We would like to highlight that we did not perform substrate-guided ablation in this study and there is still no clear understanding of the role of drivers that help to perpetuate persAF. However, in the present work we retrospectively show that different types of AF drivers might co-exist in persAF patients, and we propose a new method that helps to identify these.

### CFAEs as markers for atrial substrate in persAF

Sustained AF causes changes in the cardiac tissue characteristics, inducing structural and electric remodeling (de Bakker and Wittkampf, 2010). These regions can potentially host tissue with slow or inhomogeneous conduction, inducing re-entry circuits, resulting in fractionated fibrillatory conduction (Ashihara et al., 2012), and are important in triggering and perpetuating atrial arrhythmias. AEGs acquired from such atrial substrate regions demonstrate the low amplitude, multiple deflections activations that characterize fractionated activity.

CFAEs have been introduced as markers for atrial substrate and as targets for ablation (Nademanee et al., 2004), but failed to provide a definitive solution for persAF therapy (Oral et al., 2009; Dixit et al., 2012; Verma et al., 2015). We have recently shown that there is still no gold standard for CFAE classification in human persAF, and that CFAE target identification is dependent on the system used and settings applied during the procedure, since each system measures different aspects of the AEGs to characterize the atrial substrate (Almeida et al., 2016). Consequently, a thorough re-evaluation of the definition of CFAE is necessary to refine the identification of atrial regions responsible for the perpetuation of the arrhythmia in patients with persAF.

### Clinical inferences on LA regions resistant to PVI vs. LA regions affected by PVI

AF is a progressive arrhythmia and sustained AF might induce atrial remodeling (Wijffels et al., 1995). Understanding the substrate and the various mechanisms—and theories—of AF perpetuation is crucial to investigate the course of the arrhythmia in each patient (de Groot et al., 2016). As a consequence, the substrate of AF may require different therapy modalities at different stages. For instance, PVI alone might be sufficient at early stages, whereas atrial compartmentalization or extensive AEG-guided ablation might be necessary to terminate the arrhythmia in later stages. In the latter case, while the efficacy of CFAE-guided ablation has been questioned in recent findings (Oral et al., 2009; Verma et al., 2015), ablation targeting rotors has gained significant importance recently, although further studies are needed to consolidate its effectiveness (Narayan et al., 2012; Mohanty et al., 2016). Furthermore, even extensive ablative therapies may be ineffective in the presence of electrical dissociation between the endo- and epicardial layers of the atria, and alternative ablation strategies might be required (de Groot et al., 2016).

In the present work, we propose a technique that might help to diagnose the stage of development of the AF substrate based on multidimensional observations of the changes in the CFAE distribution in the LA induced by PVI, which is essential for patient-specific AF therapy. Previous works have shown that PVI organizes fractionation in large regions of the LA that were fractionated prior to any ablation (Roux et al., 2009; Tuan et al., 2011; Dixit et al., 2012). These findings motivated targeting CFAEs in persAF ablation just after PVI and anatomical lines. More specifically, we have recently shown that PVI+RL has changed AF dynamics from the same patients/dataset, which brings relevance to the current study (Tuan et al., 2011).

Our results also support the perception that CFAE mapping and ablation should be performed after PVI (Roux et al., 2009; Tuan et al., 2011; Dixit et al., 2012). However, our results have unveiled that there are LA regions resistant to PVI, while others are affected by it. Nearly half of the atrial regions classified as fractionated at baseline continued fractionated after PVI+RL (group 1), and most regions that were not fractionated at baseline remained organized after PVI+RL (group 4). These groups could represent atrial regions important in the perpetuation of the arrhythmia as they appear to be anchored in the atria and resistant to PVI+RL. For instance, group 1 would suggest the presence of remodeled atrial tissue with altered conduction, resulting in the fractionated activation resistant to PVI. Group 4, on the other hand, suggests the presence of organized ectopic activations or re-entries that persist even after the PVs have been isolated.

We have also identified atrial regions with fractionated signals at baseline that became organized after the ablation (group 2), and regions that were not fractionated at baseline that became fractionated after ablation (group 3). While it might be clear that atrial regions in group 1 should be targets for ablation and group 4 should not be targeted, there are some uncertainties surrounding atrial regions corresponding to groups 2 and 3. Although, it seems unlikely that these regions represent structural scar or anchored atrial substrate—as they were subjected to changes after PVI+RL—they could also represent underlying AF drivers that have moved or have been modulated by PVI+RL.

A prospective study using the method proposed in the present study—as illustrated in Figure 6B—would help to answer this question. For instance, we have recently developed an interactive platform to guide persAF ablation that performs the analysis of multiple AEG features during AF mapping, which would facilitate the implementation of the proposed method in prospective electrophysiological studies (Li et al., 2017).

### Multiple attributes for LA tissue characterization

While some might argue that this is an evidence that the attributes measured from the AEGs are not a true representation of the atrial substrate (Lau et al., 2015), most of those attributes were affected by PVI+RL, which would suggest that the attributes are measuring important electrophysiological characteristics of the atrial tissue. Individually, however, the attributes failed to discriminate the different LA region groups (groups 1 to 4), either before or after PVI+RL. The different attributes measure complementary characteristics of the AEG, and the combination of these attributes—created by multivariate statistical models—might provide a more complete characterization of the atrial tissue.

The retrospective multivariate statistical models—described here by both MANOVA and LDA—were able to discriminate the different LA region groups based on the attributes measured before and after PVI+RL. While the commercial systems had classified the LA regions only as CFAE or not CFAEs (Figure 6A), the proposed model provided further information regarding the atrial substrate (Figure 6B), classifying the LA regions into four groups (Group 1—CFAEs at baseline that remained CFAEs after PVI+RL; Group 2—CFAEs at baseline that converted to normal after PVI+RL; Group 3—normal AEGs at baseline that became CFAEs after PVI+RL; Group 4—normal AEGs at baseline that remained normal after PVI+RL).

In the present study, CFAE mapping before and after PVI+RL had to be performed in order to create the statistical models. As a result, two models have been created to discriminate the LA regions: one for baseline data, other for post-PVI data. Once created, these models could be used prospectively without the necessity of a CFAE re-map. Consequently, the prospective investigation could be performed either before or after PVI. In the first case, CFAEs collected before PVI would be used in the model created for pre-PVI data to predict which atrial regions would be resistant to PVI and which atrial regions would be affected by it. In the second case, CFAEs collected after PVI would be used in the model created for post-PVI data to estimate which atrial regions were resistant to PVI and which atrial regions were affected by it. This would allow for online characterization of different types of LA region (resistant to PVI vs. affected by it), which could help in the decision of the ablation strategy based on the multiple aspects of the atrial tissue.

### Limitations

The current study was limited to retrospective data. Further understanding of the underlying cardio-electrophysiological mechanisms behind CFAEs and the effect of PVI on the atrial substrate would be helpful for the validation of the suggested multivariate models, such as in (i) computational intracardiac models that simulate both atrial electrical activity and ablation procedures during AF (Krueger et al., 2013) and; (ii) prospective studies using the proposed models in the identification of ablation targets during substrate mapping.

Although, some patients in the present study were on anti-arrhythmic drugs in addition to ACE inhibitors and ARB, all anti-arrhythmic drugs (except for amiodarone) were stopped for at least five half-lives before the procedure. Beneficial heart failure and hypertension medications such as ACE-inhibitors and ARBs are usually not stopped for electrophysiology/ablation procedures, as the individual would invariably be taking them long-term to treat their chronic condition. We also acknowledge that amiodarone could affect fractionation of AEGs, but it is nevertheless common practice to undergo ablation whilst maintaining this drug. Our study was focused on understanding the behavior of AEG fractionation during persAF before and after PVI, therefore any resultant modulation in the underlying electrophysiological milieu due to ongoing medications would remain essentially constant over the period of data collection for each patient, and furthermore would better reflect their usual baseline clinical state. This makes the observations from our study particularly applicable to real-world practice.

The relatively small number of collected signals is a limitation of this study. This limitation, however, needs to be balanced against the facts that (i) the points have been collected from 18 patients, providing information from different LA anatomical sites and; (ii) the LOOCV approach allows for a robust model evaluation for relatively small datasets.

## Conclusions

This study retrospectively investigated changes in AEG fractionation distribution induced by PVI+RL in persAF patients using multivariate statistical models based on multiple attributes. Our results suggest that some atrial regions are resistant to PVI, while others are affected by it. While current methods for CFAE classification are insufficient to identify those different regions, the multivariate statistical models based on multiple attributes does help in the discrimination of these regions. The proposed multivariate statistical model could be considered in future electrophysiological studies for better atrial substrate characterization in persAF ablation.

## Ethics statement

All human subjects underwent standard clinical procedure that does not require approval of any ethical committee. All procedures were performed with full informed consent from the patients.

## Author contributions

TA: concept/design study, data analysis/interpretation of results, drafting manuscript, critical revision of manuscript, statistics, and “off-line” data collection; GC: data analysis/interpretation of results, critical revision of manuscript, and “off-line” data collection; XL: data analysis/interpretation of results, and critical revision of manuscript, and statistics; ND: data analysis/interpretation of results, and critical revision of manuscript; JT: EP study, data collection, interpretation of results, and critical revision of manuscript; PS: EP studies and ablation procedures, interpretation of results, and critical revision of manuscript; FS: Concept/design study, data analysis/interpretation of results, and critical revision of manuscript; GN: EP studies and ablation procedures, concept/design study, interpretation of results, and critical revision of manuscript.

### Conflict of interest statement

GA has received research fellowship from St. Jude Medical and speaker fees and honoraria from Biosense Webster. The other authors declare that the research was conducted in the absence of any commercial or financial relationships that could be construed as a potential conflict of interest.
