# Supplementary material for: Atrial Electrogram Fractionation Distribution before and after Pulmonary Vein Isolation in Human Persistent Atrial Fibrillation—A Retrospective Multivariate Statistical Analysis
Source: Front Physiol. 2017 Aug 24;8:589. doi: 10.3389/fphys.2017.00589 (PMC5573839; doi:10.3389/fphys.2017.00589)
Supplement: Supplementary file 1 [file DataSheet1.DOCX]

Supplementary Material

Atrial electrogram fractionation distribution before and after pulmonary vein isolation in human persistent atrial fibrillation – a retrospective multivariate statistical analysis

Tiago P. Almeida^1,2^, Gavin S. Chu^3^, Xin Li^1^, Nawshin Dastagir^3^, Jiun H. Tuan^4^, Peter J. Stafford^4^, Fernando S. Schlindwein^1,5^, G. André Ng^3,4,5^*

^1^Department of Engineering, University of Leicester, UK

^2^Biomedical Engineering, Center for Engineering, Modelling and Applied Social Sciences, Federal ABC University, Brazil

^3^Department of Cardiovascular Sciences, University of Leicester, UK

^4^University Hospitals of Leicester NHS Trust, UK

^5^National Institute for Health Research Leicester Cardiovascular Biomedical Research Unit, Glenfield Hospital, UK

*** Correspondence:**

Professor G. André Ng,

Department of Cardiovascular Sciences

Glenfield Hospital, Leicester, LE3 9QP

Email: gan1@le.ac.uk

Tel: +44 (0)116 250 2438 – Fax: +44 (0)116 287 5792

# Review on atrial electrogram (AEG) Attributes

## Attributes computed by commercial mapping systems

The two systems being used in clinical practice for complex fractionation atrial electrogram (CFAE) mapping are the NavX and the CARTO (Figure 1 of the main manuscript). The algorithms embedded in those systems incorporate CFAE characteristics as initially defined by Nademanee et al.,(1) and have been described in detail elsewhere.(2)

NavX computes the time interval between selected negative deflections. The negative deflections must meet some criteria to be marked to avoid noise and multiple detections, and minimize ventricular far-field interference.(2) CFE-Mean is defined as the average of the marked time intervals, while CFE-StdDev represents its standard deviation.

CARTO identifies the number of complex intervals from marked peaks and troughs of bipolar atrial electrograms (AEGs) that meet certain criteria within 2.5-s time window.(2) The number of identified complex intervals is referred to as the ICL, and characterizes the repetitiveness of the CFAE complexes. CARTO software also finds the average and shortest identified interval, referred to as the ACI and SCI, respectively.

## Information theory attributes

### Kernel Density Estimator

Probability density function (pdf) estimates are closely related to the histogram, but can be endowed with properties such as smoothness or continuity. According to Izenman (3), pdf estimates can be effective in decision making, such as nonparametric discrimination and classification analysis. The kernel density estimator (KDE) is believed to be the most popular nonparametric pdf estimation method. A pdf pattern (known as the kernel function) is summed at over N points centered at each data point *x_n_*. Hence, the summation of kernel functions centralized at each data point *x_n_* provides an estimative of the pdf of a random variable, accordingly:

$$\hat{p}\left( x \right)=\frac{1}{Nh}\sum_{n=1}^{N} K\left( \frac{x-x_{n}}{h} \right)$$

where *N* is the total number of data points, *K* is the kernel smoothing function and *h* > 0 is a bandwidth factor. Common kernel functions are Epanechnikov, Gaussian, triangular or box.(3) The choice of the bandwidth factor h is crucial in density estimation. In case a Gaussian kernel is chosen, the *h* represents the SD of the Gaussian components. Therefore, *h* is a smoothing parameter, with trade-off between sensitivity to noise at small *h* and over-smoothing at large *h* (4). In the present work, the Epanechnikov kernel smoothing function was chosen,(5) with default bandwidth defined by MATLAB. Supplementary Figure 1 illustrates the pdf of a white noise generated on MATLAB and a bipolar AEG collected during AF using the above mentioned method. The area under the curve of all pdfs calculated in this work were equal to 1, as expected.


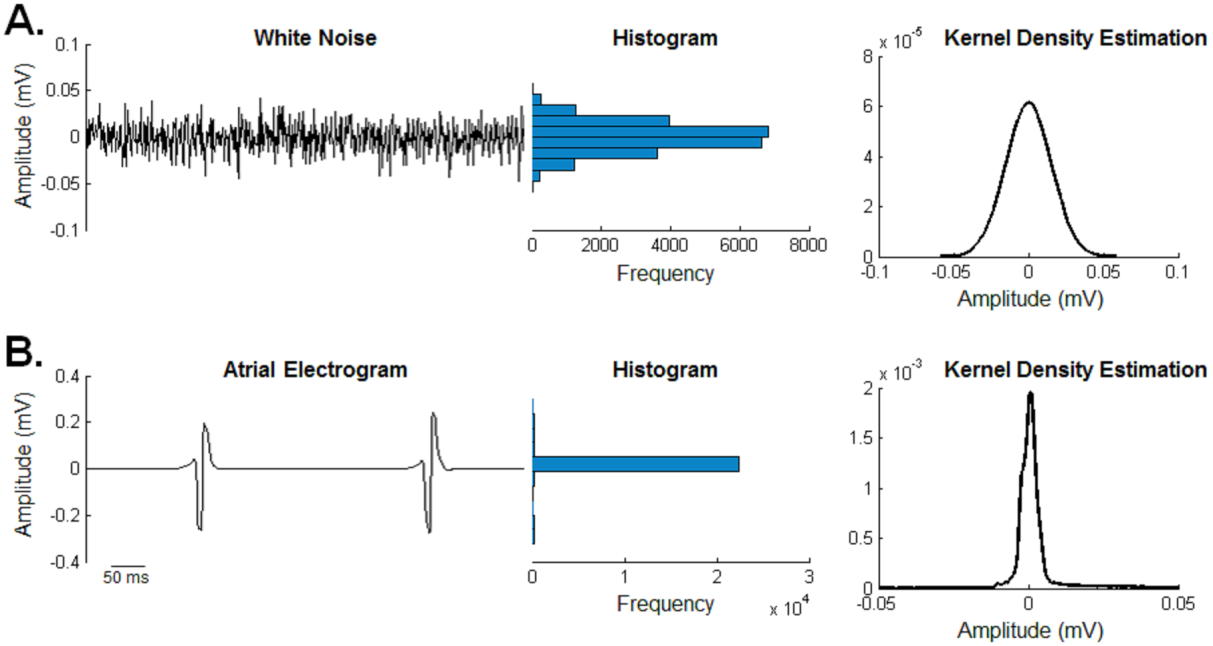


**Supplementary Figure 1.** Illustration of the estimation of the pdfs from a white noise (A) and an AEG (B) using the KDE the Epanechnikov kernel smoothing function and the default bandwidth defined by MATLAB.

### Shannon Entropy (ShEn)

The concept of entropy was first introduced by Shannon on his work about information theory as a measure of information, choice and uncertainty (6). The entropy of a random variable gives us a notion of how much information is contained in the variable. Cover and Thomas have described entropy as a measure of uncertainty of a random variable (7). The entropy H of a set of possible events (*X = x_1_, x_2_,…, x_n_*) whose probabilities of occurrence are *p_1_, p_2_,…, p_n_*, is defined by:

$$H=-\sum_{i=1}^{n} p_{i}\log p_{i}$$

The largest value of *H* occurs when the pdf has a Gaussian distribution. It assumes small values for distributions that are concentrated on certain values, or has a pdf with a sharp peak (8). ShEn is used as a measure of the degree of organization of a signal. In the present work, the probabilities of occurrences found by the KDE were used to compute the ShEn. ShEn assumes low values for AEGs with organized activation and high values for CFAEs (9). Some approximations have been proposed to calculate entropy.

### Sample Entropy (SampEn)

The Approximate Entropy (ApEn), as suggested by Pincus, is the negative natural logarithm of the conditional probability that a variable (*X*) of length *N* repeat itself for *m*+1 points after being repeated itself for m points, within a tolerance *r* (10). The similarity criterion *r* is commonly expressed as a fraction of the SD of the data, making the ApEn a scale-invariant measure. Low ApEn values indicate low complexity in the time series.

For *m* or *m* +1 points, two subsets are considered similar if

$\left| X_{i+k}-X_{j+k} \right|<r$, for 0 < k < m (or m + 1)

Consider *P_m_* as the set of all patterns from *X_N_* with length *m* (*p_m_(1), p_m_(2),…, p_m_(N-m+1)*). The fraction of patterns (*C_i,m_(r)*) of length m that resemble the pattern of the same length that begins at interval *i* can be defined by

$$C_{i,m}=\frac{n_{i,m}\left( r \right)}{N-m+1}$$

where *n_i,m_(r)* is the number of patterns in *P_m_* that are similar to *pm(i)*, given the similarity criterion of *r*. *C_m_(r)* is defined as the average of all the *C_i,m_(r)* values, which express the prevalence of repetitive patterns of length *m* in *X_N_*. The same step is repeated for *m*+1, with the constraint that *C_m+1_(r)* is a subset of *C_m_(r)* that also matches for length *m*+1. Finally, ApEn(*X_N_, m, r*) is given as

$$\mathrm{ApEn}\left( X_{N},m,r \right)=-\ln\frac{C_{m}\left( r \right)}{C_{m+1}\left( r \right)}$$

One major limitation of this method is that a correction is needed to avoid [log (0)], which has been shown as being the same as allowing templates to match themselves, which is a strong source of bias toward ApEn = 0 when there are few matches and when *C_m_(r)* and *C_m+1_(r)* are small (11).

The SampEn is the negative natural logarithm of the conditional probability that a variable (*X*) of length *N* repeat itself for *m*+1 points after being repeated itself for m points, within a tolerance *r*, without allowing self-matches (11). It has been shown that SampEn displays the property of relative consistency in situations where ApEn does not, being more robust with smaller datasets and when there are only a few matches of patterns (12). For all AEG signals, the SampEn measurements were calculated using the *r* = 0.2 and *m* = 3 (13). Similar to the ShEn, SampEn assumes high values for CFAEs.

### Kullback-Leibler (K-L) divergence

The K-L divergence, also known as relative entropy, is a measure of the distance between two distributions (7). For instance, considering that *q(x)* is an estimated distribution from an unknown distribution *p(x)*, the relative entropy *D(p||q)* is a measure of the inefficiency of assuming that the distribution is *q* when the true distribution is *p*:

$$D\left( p||q \right)=-\sum_{i=1}^{n} p_{i}\log q_{i}-\left( -\sum_{i=1}^{n} p_{i}\log p_{i} \right)$$

$$D\left( p||q \right)=\sum_{i=1}^{n} p_{i}\log\frac{p_{i}}{q_{i}}$$

The relative entropy is always non-negative and is zero if and only if *p* = *q*. In the present work, the amplitude distributions of the AEGs were compared with the amplitude distribution of a standardized Gaussian white noise, following the AEGs classification rationale proposed by Wells *et al* (14). Therefore, the K-L should assume small values for complex signals such as CFAEs.

Similar to the calculation of ShEn, K-L was calculated using the probabilities of occurrences found by the KDE.

### Amplitude based attributes

Low peak-to-peak (PP) amplitude of AEGs has been introduced as a marker for atrial scar during AF and, therefore, targets for ablation (15). However, the PP currently assessed by commercial systems refers to only one atrial activation within a time window, which might not be a good representation of the entire AEG segment. Therefore, the root mean square (RMS) of the AEG amplitude was measured considering the entire AEG segment. The RMS is the squared quadratic sum of the amplitude values in a signal *x*, i.e.:

$$x(RMS)=\frac{1}{n}\sum_{i=1}^{n} \sqrt{x_{i}^{2}}$$

Similarly, small values of RMS would suggest more fractionation.

### Frequency based attributes

The frequency domain representation of a waveform can provide more useful information than the time domain representation. Determining the frequency content of a waveform is termed spectral analysis, in which a waveform is decomposed into its constituent frequencies (16).

#### The Fourier Transform

The Fourier Transform (FT) is the most popular technique for spectral analysis. It decomposes the waveform into a series of sinusoids that are at the same frequency as, or multiples of, the waveform frequency. This family of sinusoids can be expressed either as sines and cosines, each of appropriate amplitude.

#### The Continuous FT

The continuous notation for the FT considers infinite cycle duration. Considering that

$$e^{-\frac{j2\pi}{T}}=\cos\left( \frac{2\pi}{T} \right)-\mathrm{jsin} \left( \frac{2\pi}{T} \right)$$

the Continuous Fourier Transform (CFT) of x(t) can be expressed as

$$X\left( f \right)=\int_{-\infty}^{\infty} x\left( t \right)e^{-j\frac{2\pi t}{T}}dt$$

which is the convolution between the original waveform and sines and cosines components. The absolute value of the FT |*X(f)*| is denoted as frequency spectrum of *x(t)*. The spectrum components correspond to the energy of the particular frequency component in the signal.

#### The Discrete FT & Fast FT

The equations for computing Fourier series analysis of digitized data are the same as for continuous data except the integration is replaced by summation. The waveform *x(t)* is discretized with sampling frequency *fs*, with all of the information contained in the frequency range of 0 to *fs*/2 ( *fs*/2 being the Nyquist frequency). The discrete waveform is represented as *x_k_*, where

$$x\left( t \right)=x\left( \frac{k}{f_{s}} \right)=x_{k}$$

*k* is assessed in the range of 0 < *k* < N−1, where *N* is the total number of samples. The discrete FT (DFT) is defined as

$$X\left( m \right)=\sum_{k=0}^{N-1} x_{k}e^{-j\frac{2\pi km}{N}}$$

where *m* indicates the harmonic number. The Fast FT (FFT) is a fast algorithm approach for spectral estimation based on the DFT. The computational cost required for computing the DFT of an *N*-samples waveform is proportional to *N*^2^. For a segment where *N* is an integer power of 2, the FFT algorithm reduces the computational cost to *N*log2(*N*) number of operations (17).

#### Anti-leaking Window

The absence of an anti-leaking window function is, by default, a rectangular window, which induces undesirable effects in the frequency spectrum domain due to abrupt discontinuities in the beginning or in the end of the segment (18). The edges of the waveform can induce singularity points of abrupt discontinuities that can be minimized by anti-leakage windows. The Hanning (or Hamming) anti-leakage window was used in the present work for presenting small weighting peak amplitude values of side lobes with narrow width of the main lobe peak amplitude.

#### Zero Padding

Zero-padding refers to increasing the number of samples with zero elements to improve spectral estimation representation. The zero-padding does not improve the spectral resolution, but it does improve the representation of the spectral estimation as the extra values are interpolations of the original waveform, reducing the *f_step_* in the frequency domain.

#### The Dominant Frequency (DF) and the Organization Index (OI)

The DF is defined as the biggest peak in the frequency spectrum of an AEG, and it is believed that atrial regions harboring high DF may represent sites with high periodic activation, driven by either ectopic activity or re-entry circuits (19).

Although the DF calculation from unipolar AEGs is straightforward, some pre-processing is needed for bipolar AEGs, accordingly: i) rectification; ii) low-pass filter at 20 Hz; iii) Anti-leaking Hanning window; iv) zero-padding of factor 2 or 5; v) FFT, where the DF is found (20). In the present work, DF was defined as the frequency with highest amplitude within the physiologically relevant range (3.5 to 20 Hz) (21).

The OI measures the variability of the frequency spectrum of an AEG. The OI is derived by dividing the area under the DF and its harmonics by the total power of the frequency spectrum. High OI represents less variability of frequency and, consequently, higher AEG organization (22):

$$OI=\frac{\mathrm{Area}\left( DF+harmonics \right)}{Area below frequency spectrum}$$

Supplementary Figure 2 illustrates the signal processing steps to calculate the DF and OI in a bipolar AEG.


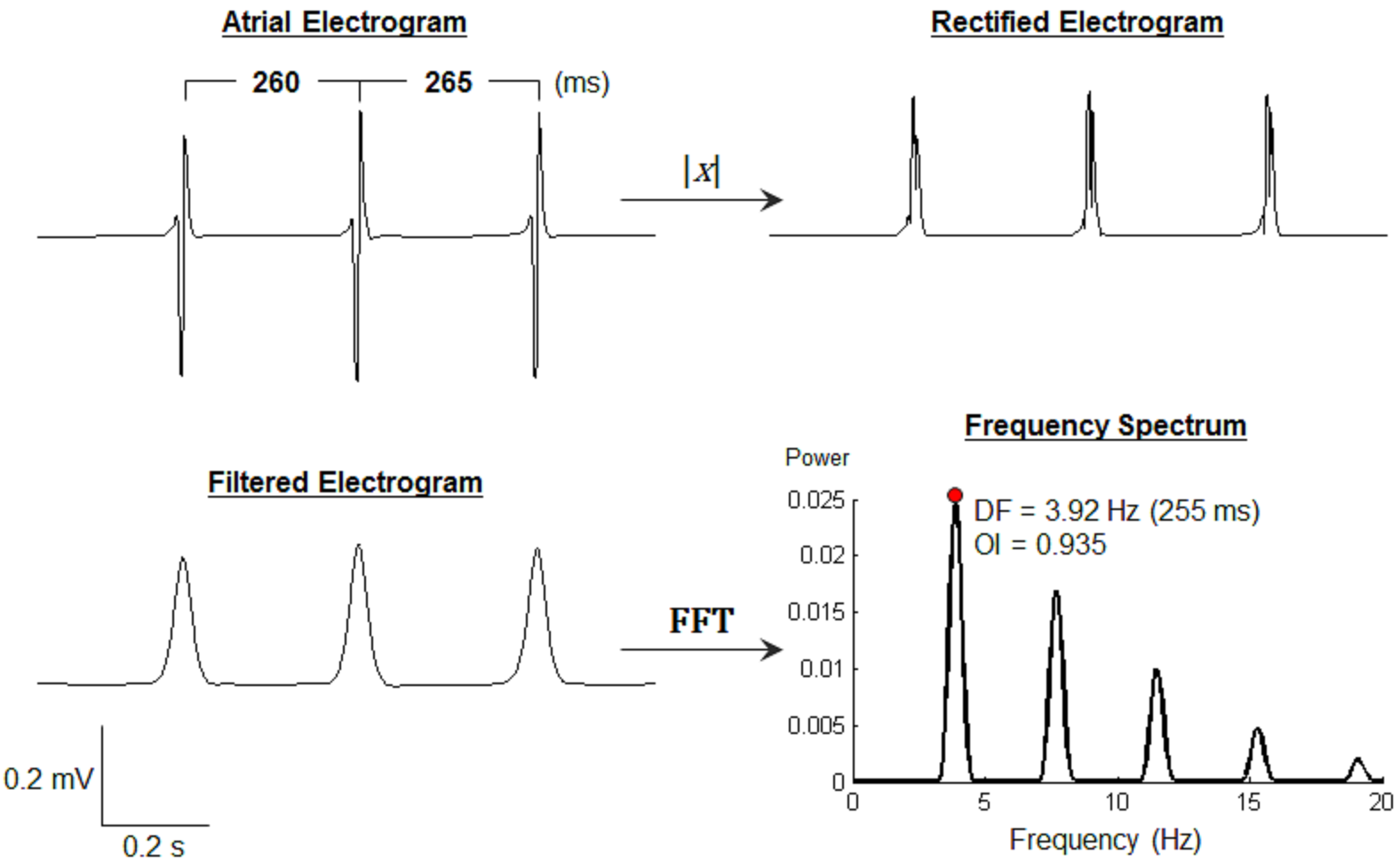


**Supplementary Figure 2.** Illustration of signal processing steps on a bipolar AEG collected during AF. A: Segment of a bipolar AEG showing 3 discrete activations with a CL of aproximately 260 ms. B: Rectified bipolar AEG. C: AEG after low-pass filtering at 20 Hz. D. corresponding power spectrum of the bipolar AEG with DF = 3.92 Hz (corresponding to a CL of 255 ms) and OI = 0.935.

# Ranked results from the MANOVA

**Supplementary Table 1**. Ranked results of effect of the group of AEGs (1, 2, 3 and 4) on the attributes before and after ablation from the MANOVA.

|  |  | Pre-ablation | | | |  | Post-ablation | | | |
| --- | --- | --- | --- | --- | --- | --- | --- | --- | --- | --- |
| AEG group |  | 1 | 2 | 3 | 4 |  | 1 | 2 | 3 | 4 |
| CFE-Mean |  | 0.476 | 0.336 | 0.395 | 0.667 |  | 0.279 | 0.622 | 0.481 | 0.559 |
| ICL |  | 0.685 | 0.563 | 0.449 | 0.218 |  | 0.790 | 0.293 | 0.451 | 0.452 |
| CFE-StdDev |  | 0.478 | 0.330 | 0.398 | 0.673 |  | 0.310 | 0.635 | 0.490 | 0.539 |
| ACI |  | 0.446 | 0.428 | 0.494 | 0.605 |  | 0.327 | 0.646 | 0.524 | 0.527 |
| SCI |  | 0.398 | 0.404 | 0.500 | 0.685 |  | 0.266 | 0.678 | 0.533 | 0.526 |
| ShEn |  | 0.497 | 0.505 | 0.482 | 0.486 |  | 0.523 | 0.487 | 0.482 | 0.487 |
| SampEn |  | 0.524 | 0.528 | 0.506 | 0.414 |  | 0.600 | 0.382 | 0.468 | 0.509 |
| KL |  | 0.505 | 0.480 | 0.502 | 0.536 |  | 0.454 | 0.542 | 0.532 | 0.514 |
| PP |  | 0.582 | 0.552 | 0.516 | 0.368 |  | 0.708 | 0.327 | 0.453 | 0.489 |
| RMS |  | 0.551 | 0.587 | 0.566 | 0.383 |  | 0.702 | 0.342 | 0.468 | 0.480 |
| DF |  | 0.537 | 0.459 | 0.497 | 0.542 |  | 0.582 | 0.534 | 0.514 | 0.394 |
| OI |  | 0.573 | 0.513 | 0.472 | 0.396 |  | 0.536 | 0.439 | 0.478 | 0.500 |

# The coefficients from the LDA

At baseline, the first discriminant function explained 89.1% of the variance, followed by 6.6% and 4.3%, successfully identifying 65% of the AEG classifications. After PVI+RL, the first discriminant function explained 97% of the variance, followed by 2.5% and 0.5%, and the correct identification of the AEG groups improved to 72%. The coefficients from the LDA can be seen in the Supplementary Table 3.

**Supplementary Table 3**. Coefficients from the LDA to discriminate the AEG groups (1, 2, 3 and 4) across the twelve attributes. Both SCI and ShEn were the attributes with the highest importance in identifying the discriminant functions before and after ablation.

|  |  | Pre-ablation | | |  | Post-ablation | | |
| --- | --- | --- | --- | --- | --- | --- | --- | --- |
| Linear discriminants |  | LD1 | LD2 | LD3 |  | LD1 | LD2 | LD3 |
| CFE-Mean |  | 1.854 | 0.072 | 3.084 |  | 0.257 | 0.701 | -0.223 |
| ICL |  | -1.862 | 0.584 | -0.918 |  | -3.230 | 0.162 | -0.803 |
| CFE-StdDev |  | -0.296 | 0.773 | -2.407 |  | 0.052 | 0.028 | 0.107 |
| ACI |  | -1.910 | -0.197 | 5.006 |  | 2.987 | 1.667 | 7.101 |
| SCI |  | 0.076 | -1.444 | **-6.722** |  | **-3.888** | 0.380 | -3.091 |
| ShEn |  | **-2.716** | **-3.444** | -2.201 |  | -3.849 | **-8.194** | **16.263** |
| SampEn |  | -0.414 | 0.3712 | 0.107 |  | -0.269 | -0.180 | -0.295 |
| KL |  | -0.096 | 0.925 | -0.015 |  | -0.624 | -0.997 | 0.231 |
| PP |  | 0.290 | 0.759 | 0.516 |  | -0.100 | 0.500 | 0.452 |
| RMS |  | -0.388 | -1.412 | -1.192 |  | 0.133 | 0.515 | 0.945 |
| DF |  | 1.160 | 0.949 | -2.019 |  | 0.141 | -3.500 | 0.861 |
| OI |  | -0.106 | -0.168 | -0.913 |  | -0.140 | -0.339 | 0.125 |

# Atrial substrate characterization before and after PVI+RL by each attribute

No single attribute on its own was able to discriminate the different LA region groups. Supplementary Figure 3 illustrates the distributions of all attributes considering the different AEG groups (1, 2, 3 and 4) at baseline. Supplementary Figure 4 illustrates the distributions of all attributes considering the different AEG groups after PVI+RL.


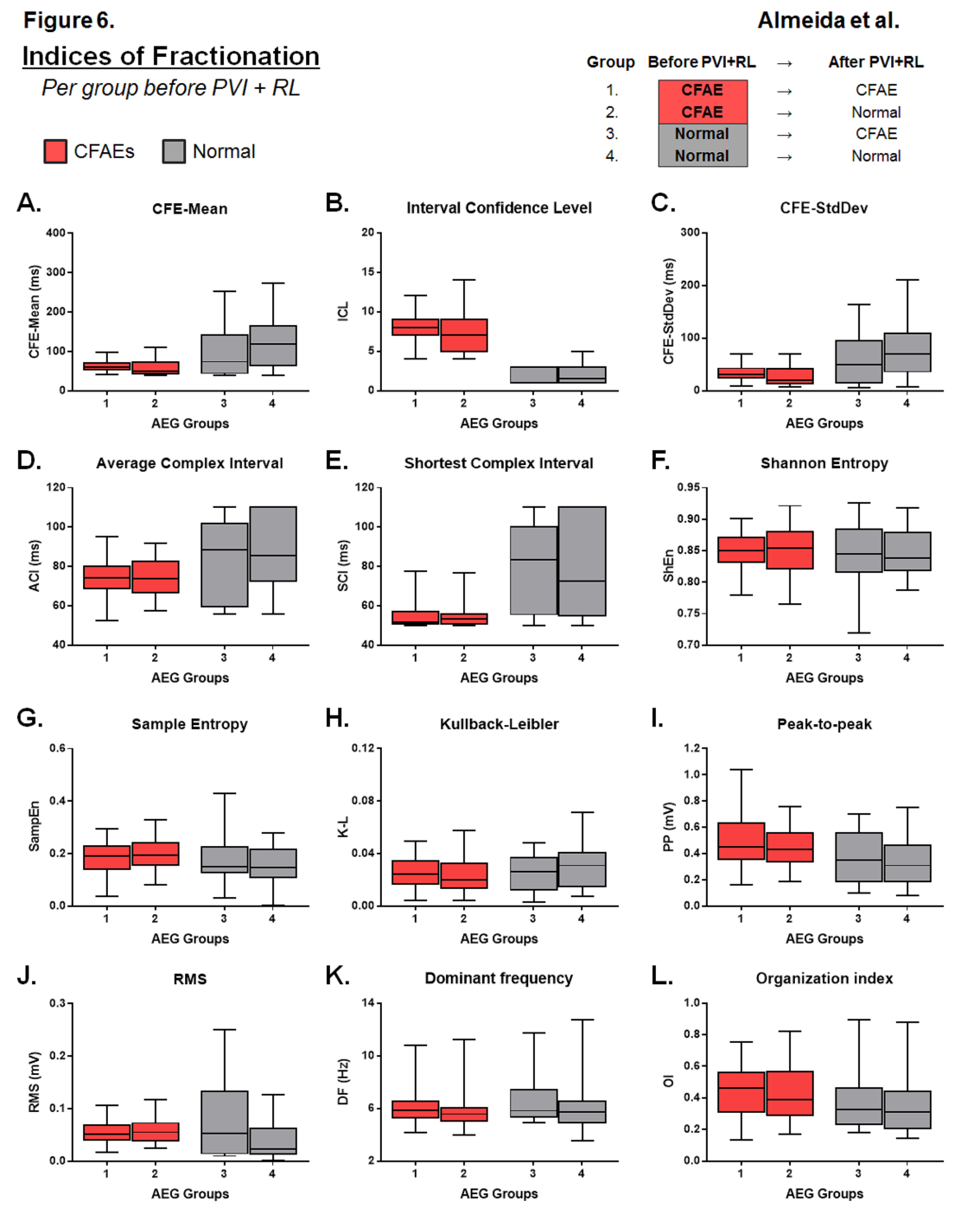


**Supplementary Figure 3.** Attributes considering the different LA regions groups at baseline – A. CFE-Mean; B. ICL; C. CFE-StdDev; D. ACI; E. SCI; F. ShEn; G. SampEn; H. K-L; I. PP; J. RMS; K. DF; L. OI.


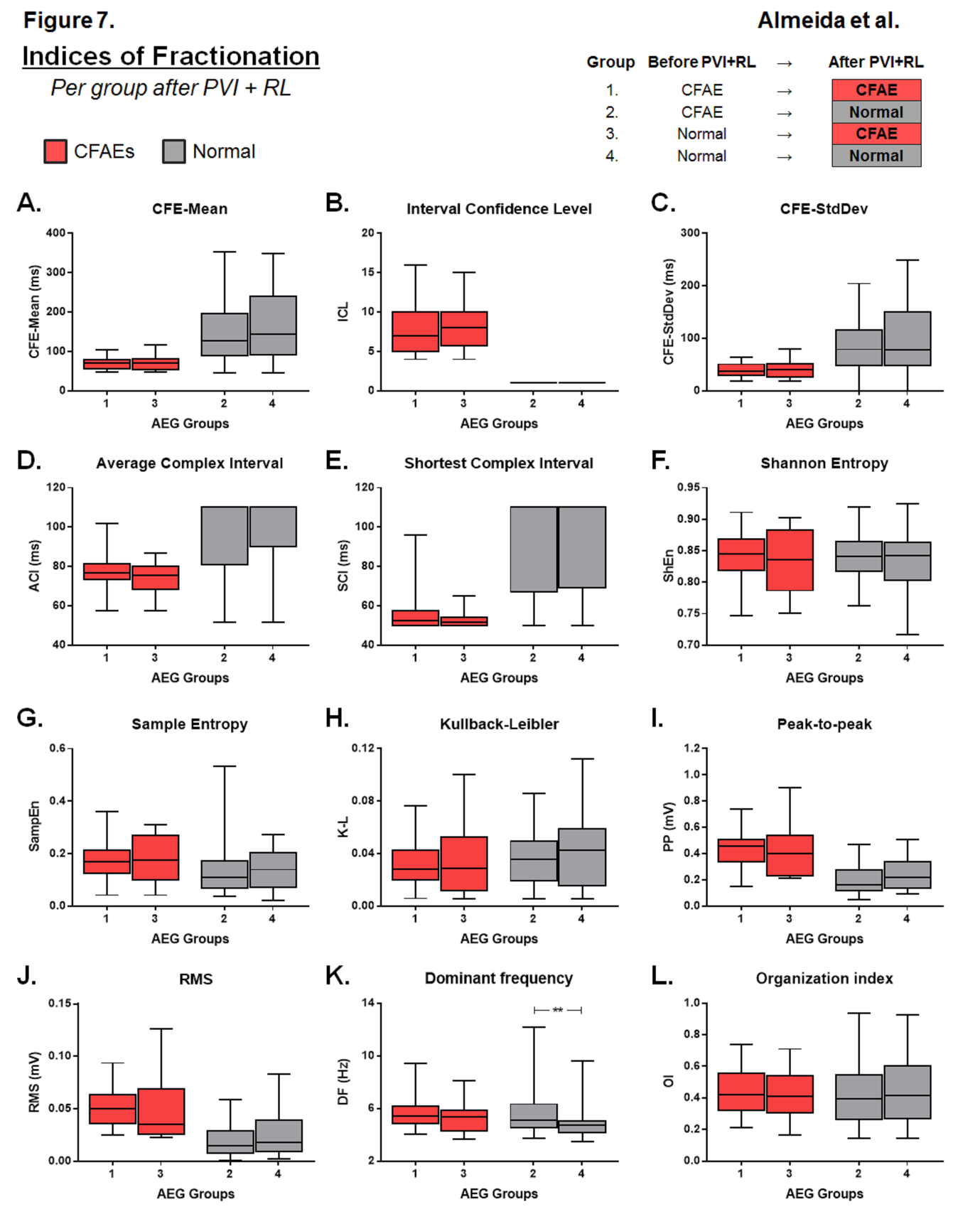


**Supplementary Figure 4.** Attributes considering the different LA regions groups after PVI+RL – A. CFE-Mean; B. ICL; C. CFE-StdDev; D. ACI; E. SCI; F. ShEn; G. SampEn; H. K-L; I. PP; J. RMS; K. DF; L. OI.

# References

1. Nademanee K, McKenzie J, Kosar E, Schwab M, Sunsaneewitayakul B, Vasavakul T, et al. A new approach for catheter ablation of atrial fibrillation: Mapping of the electrophysiologic substrate. J Am Coll Cardiol. 2004;43(11):2044-53.

2. Almeida TP, Chu GS, Salinet JL, Vanheusden FJ, Li X, Tuan JH, et al. Minimizing discordances in automated classification of fractionated electrograms in human persistent atrial fibrillation. Accepted for publication in Medical & Biological Engineering & Computing. 2016.

3. Izenman AJ. Modern multivariate statistical techniques : regression, classification, and manifold learning. New York ; London: Springer; 2008. xxv, 731 p. p.

4. Bishop CM. Pattern recognition and machine learning. New York: Springer; 2006. xx, 738 p. p.

5. Lake DE. Nonparametric Entropy Estimation Using Kernel Densities. Methods in Enzymology. Volume 467: Academic Press; 2009. p. 531-46.

6. Shannon CE. The mathematical theory of communication. The Bell System Technical Journal. 1948;27:379-423.

7. Cover TM, Thomas JA. Elements of information theory. New York: Wiley; 1991. xxii, 542 p. p.

8. Hyvarinen A, Oja E. Independent component analysis: algorithms and applications. Neural Networks. 2000;13(4-5):411-30.

9. Ng J, Borodyanskiy AI, Chang ET, Villuendas R, Dibs S, Kadish AH, et al. Measuring the Complexity of Atrial Fibrillation Electrograms. J Cardiovasc Electrophysiol. 2010;21(6):649-55.

10. Pincus SM. Approximate Entropy as a Measure of System-Complexity. P Natl Acad Sci USA. 1991;88(6):2297-301.

11. Lake DE, Richman JS, Griffin MP, Moorman JR. Sample entropy analysis of neonatal heart rate variability. American journal of physiology Regulatory, integrative and comparative physiology. 2002;283(3):R789-97.

12. Richman JS, Moorman JR. Physiological time-series analysis using approximate entropy and sample entropy. Am J Physiol-Heart C. 2000;278(6):H2039-H49.

13. Alcaraz R, Abasolo D, Hornero R, Rieta JJ. Optimal parameters study for sample entropy-based atrial fibrillation organization analysis. Computer methods and programs in biomedicine. 2010;99(1):124-32.

14. Wells JL, Karp RB, Kouchoukos NT, Maclean WAH, James TN, Waldo AL. Characterization of Atrial-Fibrillation in Man - Studies Following Open-Heart Surgery. Pace. 1978;1(4):426-38.

15. Rolf S, Kircher S, Arya A, Eitel C, Sommer P, Richter S, et al. Tailored atrial substrate modification based on low-voltage areas in catheter ablation of atrial fibrillation. Circ Arrhythm Electrophysiol. 2014;7(5):825-33.

16. Semmlow JL. Biosignal and medical image processing. 2nd ed. Boca Raton: CRC Press; 2009. xvii, 450 p. p.

17. Cooley JW, Lewis PAW, Welch PD. The Fast Fourier Transform and Its Applications. Education, IEEE Transactions on. 1969;12(1):27-34.

18. Harris FJ. On the use of windows for harmonic analysis with the discrete Fourier transform. P Ieee. 1978;66(1):51-83.

19. Sanders P, Berenfeld O, Hocini M, Jais P, Vaidyanathan R, Hsu LF, et al. Spectral analysis identifies sites of high-frequency activity maintaining atrial fibrillation in humans. Circulation. 2005;112(6):789-97.

20. Ng J, Kadish AH, Goldberger JJ. Effect of electrogram characteristics on the relationship of dominant frequency to atrial activation rate in atrial fibrillation. Heart Rhythm. 2006;3(11):1295-305.

21. Jarman JWE, Wong T, Kojodjojo P, Spohr H, Davies JE, Roughton M, et al. Spatiotemporal Behavior of High Dominant Frequency During Paroxysmal and Persistent Atrial Fibrillation in the Human Left Atrium. Circ Arrhythm Electrophysiol. 2012;5(4):650-8.

22. Everett THt, Moorman JR, Kok LC, Akar JG, Haines DE. Assessment of global atrial fibrillation organization to optimize timing of atrial defibrillation. Circulation. 2001;103(23):2857-61.
